# Clinical implementation and evaluation of a patient‐specific surface‐guided clearance mapping system for collision avoidance and noncoplanar beam planning

**DOI:** 10.1002/acm2.70548

**Published:** 2026-04-23

**Authors:** Siqiu Wang, Eric Chambers, Ruiqi Li, Yesenia Gonzalez, Xinran Zhong, Zohaib Iqbal, Kara James, Jennifer Cleaton, David Sher, Andrew Godley, David Parsons

**Affiliations:** ^1^ Department of Radiation Oncology University of Texas Southwestern Medical Center Dallas Texas USA

**Keywords:** clearance mapping, noncoplanar treatment, patient safety, surface‐guided radiotherapy

## Abstract

**Purpose/objectives:**

Collision avoidance is critical in external beam radiotherapy to ensure patient safety and plan deliverability. Limited understanding of the collision‐free treatment space risks both patient safety and unnecessary exclusion of useful beams—particularly in noncoplanar setups—resulting in suboptimal plans. Conventional methods (manual clearance checks or CT‐based assessments, etc.) are either labor‐intensive or fail to account for collision‐prone anatomy outside the scan. We investigated and clinically implemented a virtual patient‐specific clearance mapping system and evaluated its utility as a noncoplanar beam selection tool to improve plan quality.

**Materials/methods:**

The system integrates full‐body, patient‐specific surfaces—acquired during simulation using near‐infrared imaging—with interactive 3D linac/couch models. Clearance mapping accuracy was validated through phantom measurements and a comparative analysis with manual clearance checks of 60 patients across treatment sites. Workflow efficiency data were reported over three years of clinical implementation. A workflow for patient‐specific non coplanar beam selection was proposed and evaluated in 20 lung stereotactic body radiation therapy (SBRT) and 18 breast stereotactic partial breast irradiation (sPBI) cases.

**Results:**

The clearance mapping accuracy was within ± 1° (gantry/couch rotation) of phantom measurements. For 60 patients, the virtual predictions accurately identified all potential clearance issues, while manual verification missed 5 collision events. Virtual checks saved approximately 15 min of linac and therapist time per plan and eliminated an average 6.2‐clinical hour planning delay. With the proposed beam selection workflow, noncoplanar replans for lung SBRT improved target conformality (Paddick Conformity Index from 0.89 to 0.91, *p* < 0.01) and reduced low dose spillage. For breast sPBI, heart mean dose was lowered (103 cGy to 68 cGy, *p* < 0.01). Delivery time increased by approx. 30s per plan.

**Conclusions:**

The virtual clearance mapping system outperformed manual verification, streamlined clinical workflow, and could significantly improve plan quality through efficient noncoplanar beam selection. It has replaced manual verification at our institution.

## 1 Introduction

Surface‐guided radiotherapy (SGRT) has been widely applied in radiation oncology clinics around the world for patient setup and motion monitoring as an “independent observer” in the treatment room.^1^ SGRT is shown to improve the treatment delivery workflow as well as overall patient safety.^2^ In recent years, researchers and vendors have begun to further explore SGRT to develop tools for a variety of new applications throughout the radiotherapy process, including markerless setup and tracking, biometric patient identification, synergy with adaptive radiotherapy, and clearance mapping.^3^

Collision avoidance is a critical component of patient safety and plan deliverability in external beam radiotherapy. Modern planning techniques increasingly utilize additional degrees of freedom—such as collimator rotation, gantry angle, and couch angle—to optimize the dose distribution. However, this added flexibility comes with an elevated risk of mechanical collisions. In particular, conventional C‐arm linear accelerators (linacs) achieve noncoplanar delivery by introducing nonzero couch angles, which can significantly constrain the usable beam space due to gantry–couch and gantry–patient collision risks.^4^ As a result, despite the potential dosimetric benefits of noncoplanar beams—such as reduced volumetric lung and heart doses for lung stereotactic body radiation therapy (SBRT)^5^, ^6^, ^7^ and critical organ sparing for breast treatments,^8^, ^9^—clinicians often avoid such configurations due to the lack of precise, patient‐specific clearance mapping tools. Even when a noncoplanar arrangement is employed due to clinical necessity, planners often make conservative estimates of the collision space based on their experience, eliminating potentially useful beam angles in a substantial number of cases.^10^ This approach is suboptimal for personalizing plans and quality. While full beam‐space optimization, that is, 4π optimization, has yet to become standard clinical practice, studies like Sheng *et al*. have shown that plan quality can be meaningfully improved via simple noncoplanar beam arrangement when the planners are equipped with a better understanding of the clearance space.^10^

Many clinics implement manual clearance verification procedures (e.g., therapist‐led dry run with treatment devices only or with the patient) that are time‐consuming and may not fully account for patient‐specific anatomy. Virtual methods have been proposed over the years to reduce this resource burden. Earlier works tend to either focus only on gantry/couch collisions^11^ or utilize simple geometry to represent a generic patient.^12^, ^13^ In pursuit of more individualized collision avoidance strategies, recent studies made use of the patient's body from simulation CT scans,^14^, ^15^ which often do not include parts of the patient anatomy that are the main culprits of collision, for example, elbows. SGRT presents an optimal alternative to address this issue. Experimental proof‐of‐concepts and in‐house solutions have been proposed to combine patient surfaces captured by stereoscopic cameras or 3D light detection and ranging (LiDAR) scanners with virtual 3D machine models to predict collision.^16^, ^17^, ^18^, ^19^ Recently, a commercial virtual clearance mapping system (MapRT; Vision RT Ltd., London, UK) using patient‐specific surface and interactive machine models was made available and its technical performance was benchmarked.^20^

In this work, we present a methodology to evaluate the accuracy of the virtual clearance mapping system and share insights on treatment planning workflow efficiency improvement from over three years of clinical testing and implementation. Furthermore, we propose a clinically feasible beam selection workflow using this system to enable the efficient integration of noncoplanar beam arrangements and enhance overall treatment plan quality.

## 2 Materials and Methods

The MapRT system utilizes whole‐body patient‐specific surfaces, acquired during simulation via near‐infrared light‐based surface imaging, and linac and couch models built from 3D LiDAR scans for collision prediction. The system performance was evaluated via phantom study, validation against therapist‐led manual dry runs, and timing and workflow efficiency analysis. To further explore the system's utility, we proposed a noncoplanar beam selection workflow and demonstrated its potential to enhance plan quality through a retrospective planning study.

### 2.1 Brief description of the system

**FIGURE 1:**
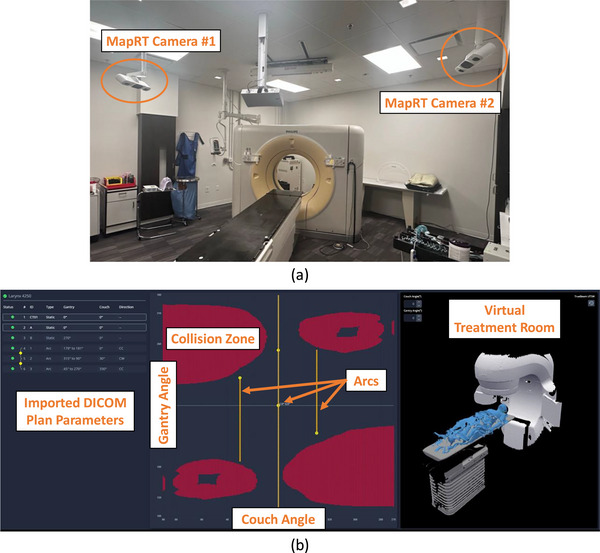
(a) The MapRT camera setup in the CT vault at our institution and (b) the interactive MapRT clearance mapping system interface.

MapRT is a commercially available virtual clearance mapping system that has recently received clearance from the U.S. Food and Drug Administration. Our institution has installed and evaluated a prototype version of the system over the past three years as part of a clinical testing effort. The hardware component enabling patient surface capture consists of two wide‐field stereoscopic camera pods situated diagonally in the vault on each side of the simulation CT scanner (Figure 1a). The pods project a speckled light pattern, establishing a set of spatial coordinates in the targeted area, which is subsequently captured by the stereoscopic cameras and processed to facilitate the reconstruction of a 3D surface. The MapRT surface acquisition software is installed on a workstation in the CT vault. DICOM RT plan files can be imported to MapRT, enabling calculation of the clearance map (for a map of gantry vs couch angles that avoid collisions) for the specific patient, linac, and couch. (Figure 1b). A “virtual treatment room” within the web browser‐based software allows the gantry and couch to be manipulated by the planner to select optimal beams in real time.

### 2.2 Phantom validation

The accuracy of the clearance mapping system was initially verified using phantom measurements. To simulate a clinical scenario with potential collision risks, the VisionRT stereotactic cube phantom (Vision RT Ltd., London, UK) was positioned at a superior‐lateral corner of the simulation CT couch and a CT scan was acquired. The phantom's surface was captured using the system's surface capture component at CT and stored in the system database.

A test plan was created in the treatment planning system using the acquired CT scan and subsequently exported to the clearance mapping system. A series of couch and gantry angle combinations near the collision threshold—beyond which gantry and phantom were predicted to collide by the mapping system—were tested in the treatment vault with the phantom placed in the same superior‐lateral couch corner. For each combination, the gantry was advanced toward the phantom at 0.1° increments until it reached a near‐collision state, defined as approximately 1 mm of clearance without contact. The corresponding gantry and couch angles were recorded as the physical collision threshold for validation.

### 2.3 Comparison with manual dry runs

During the initial implementation phase of the prototype system, patient‐specific surface data were acquired for 60 clinical cases (Varian: *N* = 33, Elekta: *N* = 27) involving various SBRT and cranial treatment sites, for which manual dry runs remained the primary method of clearance verification. Each treatment plan was retrospectively evaluated using the virtual clearance mapping system. The clearance predictions from the system were then compared to the outcomes of the corresponding manual dry runs. Additionally, the processing and turnaround times associated with both the manual and virtual clearance workflows were recorded and analyzed for comparison.

### 2.4 Noncoplanar beam selection workflow

**FIGURE 2:**
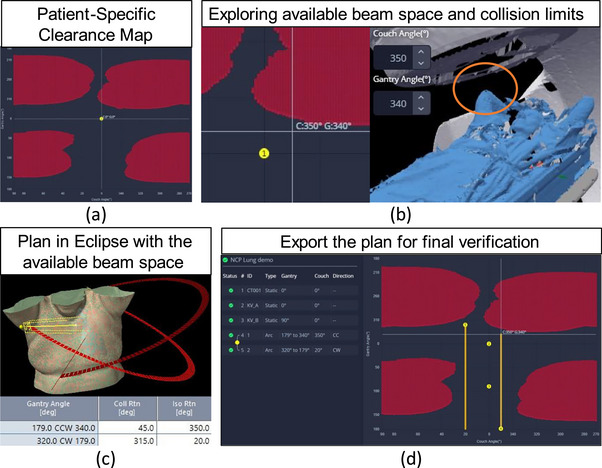
Noncoplanar beam selection workflow: (a) generate the patient‐specific clearance map using the patient surface captured at simulation and the treatment machine model, (b) explore the clearance map in the interactive system to find the collision limits, with special attention to collision‐prone anatomy (e.g., the elbow), (c) create a treatment plan using the beam clearance knowledge, and (d) send the plan to the system for a final verification.

To evaluate the potential planning benefits of the clearance mapping system as a practical tool for noncoplanar beam selection, 20 lung SBRT and 18 breast stereotactic partial breast irradiation (sPBI) cases that were originally planned with coplanar VMAT arcs (standard practice in our clinic) were replanned using a noncoplanar VMAT arc arrangement. Noncoplanar beam geometries were selected interactively with MapRT (Figure 2). The replans used the same planning objectives as the original plans to enable a fair comparison. Statistical significance between the coplanar and noncoplanar plans was determined using Student's *t*‐test. Furthermore, to assess the delivery time difference between the coplanar and noncoplanar plans, 5 plans for each site were randomly selected and delivered on a clinical linac (without patients).

## 3 Results

### 3.1 Phantom study

**FIGURE 3:**
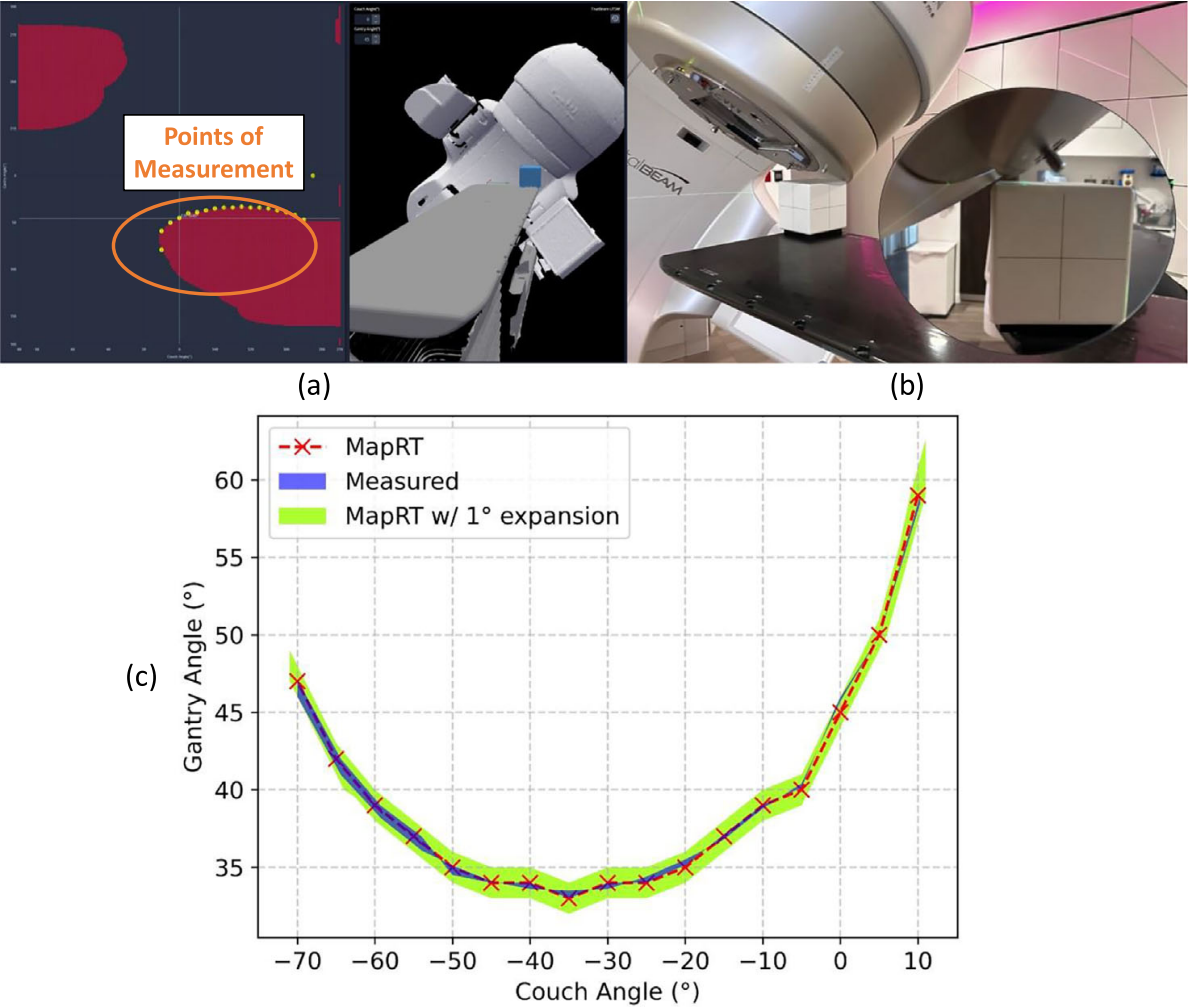
(a) Setup of the phantom measurement in the clearance mapping system and (b) on the treatment couch. (c) Phantom/gantry collision limits by phantom measurement vs. virtual clearance mapping system prediction (and prediction with 1‐degree gantry/couch angle).

Seventeen combinations of gantry and couch angles (33°–59° and ‐70°–10°, respectively) were analyzed and compared. The phantom measurements and the system‐predicted collision limits agreed within ± 1° of gantry/couch rotation (Figure 3).

### 3.2 Comparison with manual dry runs

The clearance results for the 60 cases that received both manual and virtual clearance checks during the initial implementation phase of the prototype system are shown in Table 1. Collision events were identified either during the pretreatment clearance check with the patient positioned on the treatment couch or during the treatment session itself; in both scenarios, a replan and the subsequent delay were required to proceed safely. The cases were collected from departmental quality reviews and/or internal communications.

**TABLE 1:** Collision detection accuracy of the manual dry run vs. the clearance mapping system. *Positive: True and false positive events could not be separately identified in manual dry run, as the plans with potential collision risks would not have proceeded to treatment.

Method	Positive*|TN|FN	Success ratio
Manual dry run	5|50|5	91.7%
Clearance mapping system	10|50|0	100%

The clearance mapping system was able to correctly identify all positive occurrences (100% success ratio), while the manual dry runs missed 5 collision events that were later discovered at treatment. It should be noted that the true positive and false positive events cannot be separately identified, as the plans would not have been cleared for treatment.

**FIGURE 4:**
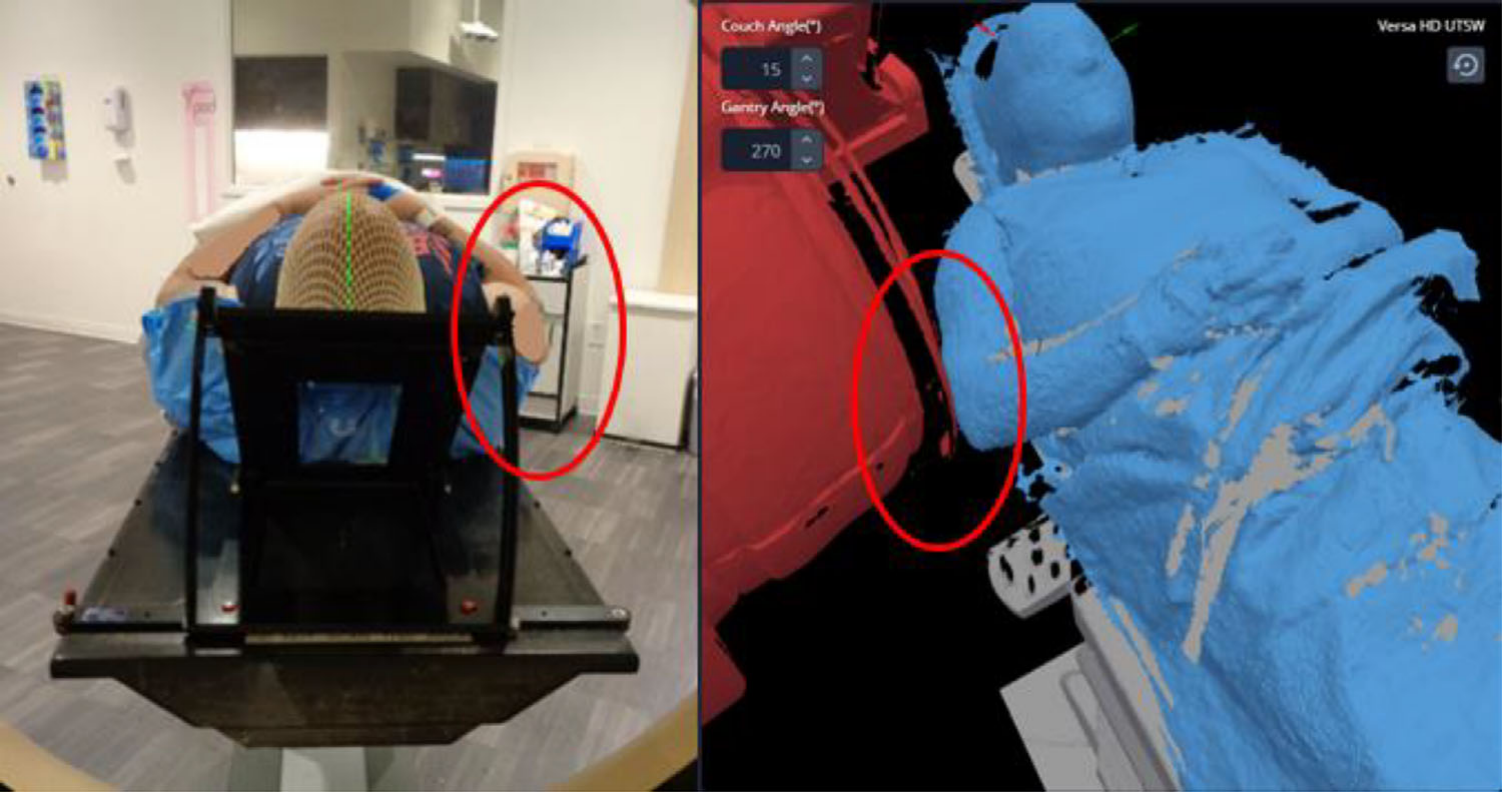
The patient setup at simulation (left) and the surface capture in the clearance mapping system (right). The gantry being red indicates a collision event (in this case with the patient's arm).

An example of a false negative collision event missed by a manual dry run is shown in Figure 4, where the patient's arm collided with the gantry safeguard. This would not have been detected until treatment without the patient‐specific surface.

### 3.3 Efficiency and workflow considerations

**FIGURE 5:**
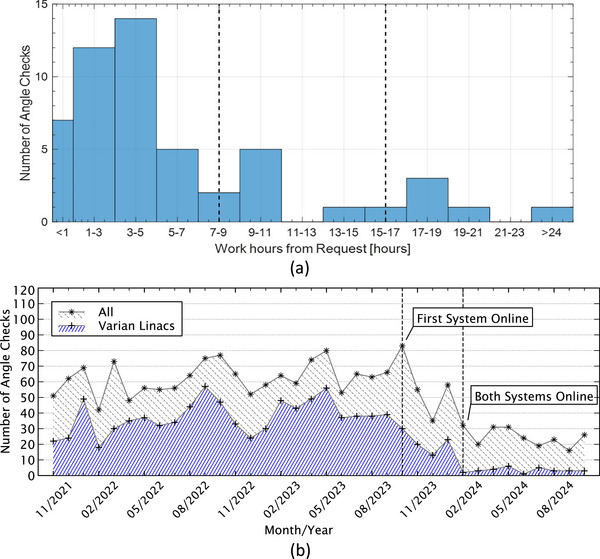
(a) Histogram of the wait time (in clinical hours) between manual dry run request and completion for the 60 evaluated cases. (b) Number of manual angle checks performed per month over time on all C‐arm linacs in our clinic.

Each manual dry run at our institution occupies 15 min on average of machine and therapist time. More critically, the feasibility of completing a dry run is contingent upon the timing of the request from the planner and the availability of both the linac and therapist, resulting in turnaround times ranging from 0.35 to 37.57 clinical hours among the 60 investigated cases (Figure 5a). This is further exacerbated by instances where modifications to the plan necessitate a second dry run, based on the initial dry run outcome.

In comparison, surface capture at simulation only adds one min to the workflow. The planner can then complete the virtual clearance mapping process remotely in less than 5 min via the web interface. Furthermore, should the initial plan fail to clear, the planner can adjust the beam parameters while checking clearance in real time, avoiding a repeat of the manual dry run with its further delays.

Over the three years of clinical integration of the two virtual clearance mapping systems at both simulation CTs, the number of manual angle checks performed in our institution has decreased significantly (Figure 5b). Our institution has both Elekta and Varian linacs. While the Varian machine models came ready with the system, the Elekta ones only recently finished development. A similar reduction in manual clearance checks is anticipated following its clinical deployment.

### 3.4 Noncoplanar planning

**FIGURE 6:**
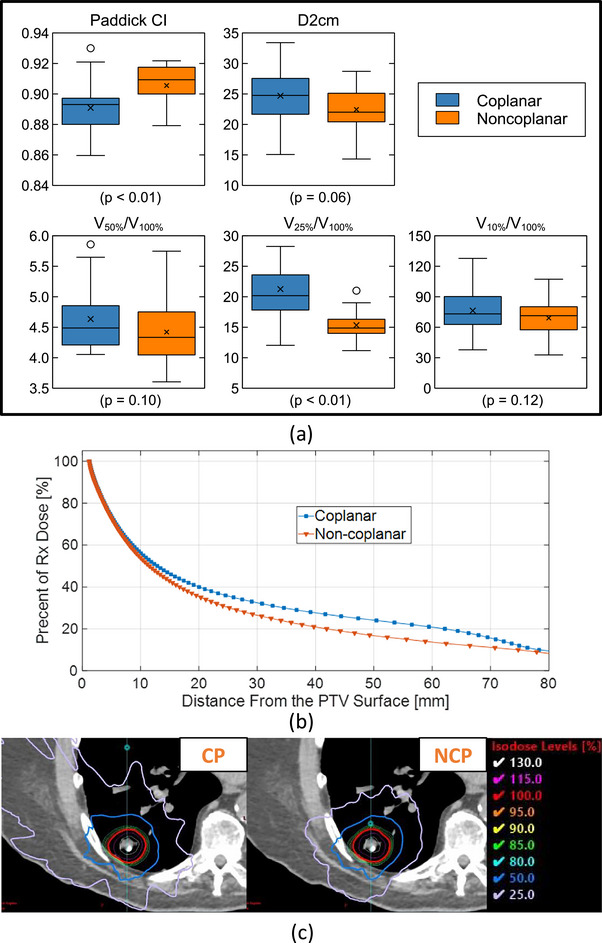
Plan quality metrics (a) and dose falloff from target surface plot (b) and an example (c) of the coplanar (CP) vs. noncoplanar (NCP) lung SBRT plans.

**FIGURE 7:**
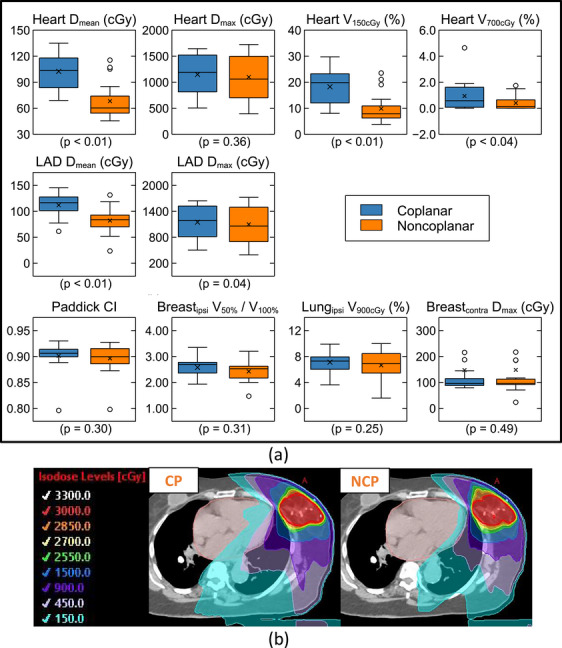
(a) Heart and left anterior descending artery (LAD) doses, and other plan quality indices, and (b) an example of the coplanar (CP) vs. noncoplanar (NCP) breast sPBI plans.

All plans used two isocentric partial VMAT arcs (Figures 6 and 7). For lung SBRT, our typical noncoplanar beam arrangement uses two arcs separated by approximately 30° couch rotations (e.g., one at 350° and the other at 20°), with the specific couch angles adjusted as needed based on the patient‐specific clearance space predicted by MapRT. For breast sPBI, the beam angles were selected based on the target's relative position to the heart, the main OAR considered, so a wider range of couch rotations up to 270° was used.

For lung SBRT, plan quality indicators, that is, Paddick conformity index (CI_Paddick_) (measures how well the prescription‐dose volume matches the target), maximum dose at a distance ≥2 cm away from the PTV (d2cm), and gradient indices,^21^ were calculated and compared between the coplanar and noncoplanar arc replans (Figure 6a). The d2cm and gradient indices quantify the lower dose falloff away from the target. The noncoplanar beam angles selected via the clearance mapping system significantly improve the target conformity (CI_Paddick_: 0.89 ± 0.02 (coplanar) and 0.91 ± 0.01 (noncoplanar) with *p*‐value = 0.001) and the gradient index, especially at 25% prescription dose (Gradient Index: 21.3 ± 4.4 (coplanar) and 15.3 ± 2.2 (noncoplanar with *p*‐value < 0.001)). Overall, the noncoplanar replans exhibit favorable dose falloff at a wide range of distances from the PTV surface (Figure 6b).

For breast sPBI, additional OAR mean, max, and volumetric doses were calculated and compared (Figure 7). While maintaining similar conformity, contralateral breast and ipsilateral lung doses, the noncoplanar replans significantly (*p* < 0.05) reduced the mean and volumetric doses to the heart and the left anterior descending artery (LAD). The maximum dose for the heart and LAD was unchanged between the two techniques.

The noncoplanar treatment plans required, on average, an additional 26.4 s to deliver compared to coplanar plans (164.5 ± 20.6 s with a median of 152 s vs. 148.1 ± 26.9 s with a median of 135 s, respectively). In accordance with our institutional safety policy, couch rotation was performed inside the vault after each beam, which primarily contributed to the extended delivery time.

## 4 Discussion

A comprehensive evaluation was conducted for a commercially available patient‐specific surface‐guided clearance mapping system, MapRT. The mapping accuracy was found to be within 1° gantry/couch rotation of physical measurements with a phantom. The system outperformed the traditional manual dry run method in successfully identifying potential collision events while significantly reducing clearance check turnaround time to optimize the radiotherapy planning workflow efficiency. Furthermore, the clearance maps enabled noncoplanar beam arrangement with minimal collision risks and improved target conformality and dose falloff for lung SBRT and significantly reduced the volumetric doses to the heart and LAD for sPBI. This is particularly important as every 1 Gy increase in mean heart dose has been correlated to an excessive 7.4% increased risk of major coronary events in the patient's lifetime, even at low radiation doses.^22^, ^23^

Previous studies on collision avoidance using patient‐specific surface guidance established the foundation for this commercial prototype. Yu *et al*.^17^ and Padilla *et al*.^16^ proposed the mechanism of patient‐specific surface capture using 3D optical scanning systems and constructed highly accurate 3D treatment machine models, while Cardan *et al*.^18^ proceeded to construct a general collision mapping framework for clinical applications with real patients. Islam *et al*.^19^ further optimized the collision detection algorithm. Sheng *et al*.^10^ recently presented a planning study utilizing a single MapRT surface capture for 8 plans and noted that potential collisions were correctly identified only 89% of the time by planners based on their experience, and 100% when using MapRT. To our knowledge, MapRT is the first commercially available product integrating SGRT into the treatment planning workflow for patient‐specific clearance mapping, and this work constitutes the first extensive clinical evaluation designed to quantify its impact on the treatment plan workflow efficiency and quality.

There are limitations to the study methodology as well as to the system in its current state. First, in the phantom study, the near‐collision gantry and couch angles were acquired by bringing the gantry and couch as close as possible to the phantom without causing a real collision. The distance and angle to the actual collision threshold were estimated to be < 1 mm and < 0.2° gantry rotation. This minor uncertainty is unlikely to cause issues in clinical operations, as a user‐specified safety buffer around the patient surface and treatment couch (e.g., 2 cm) is recommended to accommodate minor patient motion and to ensure patient safety. Secondly, the surface capture system can pick up draped linen near the patient and erroneously identify them as sources of collision risks. In practice, simulation therapists should be trained to optimize conditions for surface capture and minimize linen that hangs off the patient. Surfaces can be post‐processed offline using publicly available 3D mesh editing tools if needed. In general, any identified collision should be carefully reviewed to ensure its validity.

While the noncoplanar replans already demonstrated enhanced quality, the selection of beam arrangements was subjectively determined by the planner based on their experience. For future endeavors, incorporating the patient‐specific clearance maps into beam arrangement optimization algorithms,^6^, ^24^ especially using a forthcoming Automated Programming Interface (API) in MapRT for connection to treatment planning systems, presents an opportunity to systematically achieve personalized optimal plan quality.

## 5 Conclusion

The virtual patient‐specific surface‐guided clearance mapping system outperformed manual dry runs in identifying clearance issues. Clinical implementation of the virtual system ensures patient safety, improves planning workflow efficiency, and facilitates safe and personalized noncoplanar beam selection, with the potential for significant dosimetric benefits.

## AUTHOR CONTRIBUTIONS

Siqiu Wang and David Parsons conceptualized and designed the study. Siqiu Wang led the data collection/analysis and the manuscript writing. Ruiqi Li, Xinran Zhong, Yesenia Gonzalez, and Zohaib Iqbal contributed to planning data collection and methodology development. Eric Chambers, Kara James, and Jennifer Cleaton assisted with clinical data collection and workflow evaluation. Andrew Godley and David Sher contributed clinical expertise, evaluation of clinical significance, and critical manuscript revisions. David Parsons supervised the project, assisted with data collection/analysis, and provided critical manuscript revisions. All authors reviewed and approved the final manuscript.

## CONFLICT OF INTEREST STATEMENT

Siqiu Wang, Xinran Zhong, and David Parsons have received travel and accommodation support to present relevant research findings at scientific conferences. All other authors have no conflicts of interests to report. The MapRT system used in this work was provided by Vision RT Ltd. for clinical testing, but the company had no role in study design, data analysis, or manuscript drafting.
